# Effects of Feature-Based Explanation and Its Output Modality on User Satisfaction With Service Recommender Systems

**DOI:** 10.3389/fdata.2022.897381

**Published:** 2022-05-06

**Authors:** Zhirun Zhang, Li Chen, Tonglin Jiang, Yutong Li, Lei Li

**Affiliations:** ^1^Department of Computer Science, Hong Kong Baptist University, Kowloon, Hong Kong SAR, China; ^2^School of Psychological and Cognitive Sciences and Beijing Key Laboratory of Behavior and Mental Health, Peking University, Beijing, China; ^3^Social Science Division, University of Chicago, Chicago, IL, United States

**Keywords:** recommender system (RS), user satisfaction, feature-based explanations, output modality, user study, service product domain

## Abstract

Recent advances in natural language based virtual assistants have attracted more researches on application of recommender systems (RS) into the service product domain (e.g., looking for a restaurant or a hotel), given that RS can assist users in more effectively obtaining information. However, though there is emerging study on how the presentation of recommendation (vocal vs. visual) would affect user experiences with RS, little attention has been paid to how the output modality of its *explanation* (i.e., explaining why a particular item is recommended) interacts with the explanation content to influence user satisfaction. In this work, we particularly consider *feature-based explanation*, a popular type of explanation that aims to reveal how relevant a recommendation is to the user in terms of its features (e.g., a restaurant's food quality, service, distance, or price), for which we have concretely examined three content design factors as summarized from the literature survey: *feature type, contextual relevance*, and *number of features*. Results of our user studies show that, for explanation presented in different modalities (text and voice), the effects of those design factors on user satisfaction with RS are different. Specifically, for text explanations, the number of features and contextual relevance influenced users' satisfaction with the recommender system, but the feature type did not; while for voice explanations, we found no factors influenced user satisfaction. We finally discuss the practical implications of those findings and possible directions for future research.

## 1. Introduction

In the era of Internet, it is not uncommon to see that people search online for relevant information that could help them make decisions ranging from what to eat for dinner, to which house is appropriate for them to buy. With the explosion of information, it is critical for them to efficiently identify which information is relevant to their interests, thus resulting in the emergence and rapid development of recommender systems (RS), since they can effectively eliminate online information overload by assisting users in locating items they need (Ricci et al., 2015). More notably, with recent advances in natural language based virtual assistants, recommender systems have become popular in those commercial systems (e.g., Amazon Alexa^1^ and Apple Siri Suggestions^2^) for providing dedicated decision support. In consequence, there is emerging study on evaluating user experiences with such RS applications, especially regarding the way of how recommendations are presented. For instance, Yang et al. (2018) compared the user behaviors on vocal and visual podcast recommender systems and found that users were less efficient to consume, explored less items, and focused more on top-ranked items in the vocally presented recommendations than in the visual condition. Kraus et al. (2019) surveyed a focus group of e-commerce experts on their experiences in the voice and web commerce recommenders and found that convenience has a larger effect in the voice commerce. Zhang and Yang (2020) considered the shopping behaviors on vocal as distinct from visual when they designed a network-based recommender system, which include the tendency to consume more low-consideration products and purchase repeatedly. However, the effects of the output modality of *recommendation explanation* on user experience have rarely been investigated.

Indeed, in the area of recommender systems, explanation has broadly been recognized as an important component in addressing the “black box” phenomenon of traditional RS (Tintarev and Masthoff, 2012; Nunes and Jannach, 2017). It has been found that providing explanation for the recommendation can be helpful for not only enhancing the recommendation's transparency, but also increasing user satisfaction with the system (Herlocker et al., 2000; Sinha and Swearingen, 2002; Tintarev and Masthoff, 2012; Zhang and Chen, 2020). In existing RS, explanation is normally given in *natural language* (NL; Nunes and Jannach, 2017). In comparison with other formats such as visual charts or diagrams, NL style explanation is more persuasive as perceived by users (Kouki et al., 2019). Moreover, its usage in virtual assistants is distinct, since the delivery of NL explanation is natural in such settings (Yang et al., 2018). It hence raises the question of *whether* and *how* the **output modality** (e.g., text vs. voice) of a NL explanation would influence user satisfaction with RS, and furthermore how the output modality would interact with content design factors of the explanation to take the effects.

In this work, we have been engaged in answering the above questions. In particular, among various types of NL explanation that have been proposed so far (Herlocker et al., 2000; Chen. and Pu., 2005; Zhang et al., 2014; Lu et al., 2018), we mainly consider the ***feature-based explanation***, because it is proven being more effective than the traditional user-based and item-based explanations by providing richer, multifaceted information about the item to support users to make more informed and accurate decisions (Bilgic and Mooney, 2005; Tintarev and Masthoff, 2015; Chen and Wang, 2017; Chen et al., 2019a; Zhang and Chen, 2020). Specifically, the feature-based explanation contains one or more features of the currently recommended item in the current user's favor, e.g., “Item X is recommended because it contains feature a, b, …, which are included in items Y, Z, …, that you have already rated” (Symeonidis et al., 2008, p. 1265). It has been popularly developed in various product domains, especially for service products because it shows that users' decision-making is largely contingent on the product's features (e.g., the restaurant's food quality, service, distance, price, etc.) (Zhang et al., 2014; Chen and Chen, 2015).

The literature survey of related work on feature-based explanations reveals that they mainly vary in terms of three content design factors (Gönül et al., 2006; Chen and Pu, 2010; Chen and Chen, 2015; Sato et al., 2018): *feature type, contextual relevance*, and *the number of features*. Feature type refers to the type of feature to be explained, e.g., whether it primarily emphasizes the *positive* feature(s) of the recommended item (e.g., “delicious food” of a restaurant), or accommodates both *positive* and *negative* ones (called two-sided, e.g., “delicious food, *but* high price”). Contextual relevance is about whether the explanation will disclose the item feature's relevance to the user's current context or not (e.g., “the atmosphere is relaxing for family dinner”). The number of features indicates how many features are contained in the explanation (also called explanation length, e.g., “delicious food” that contains a single feature, while “delicious food and friendly service” contains two features).

It is then interesting to study *whether* and *how* these three content factors would interact with the explanation's output modality (text or voice) to affect user satisfaction with the recommender system. With this objective, we have concretely conducted two user studies^3^ on a restaurant RS. Specifically, in the first (pilot) study that was conducted in a controlled lab setting, we mainly investigated whether those three content design factors, i.e., feature type, contextual relevance, and the number of features, would actually take effects on user perception of an explanation's effectiveness (i.e., whether the information provided in the explanation is sufficient for users to assess the recommended item; Pu et al., 2011) or not. Motivated by the first study's findings, we further performed a formal study in an online setting, by which we not only measured the effects of feature-based explanations on user satisfaction with the recommender system, but also tested how the effects would be moderated by the explanation's output modality (i.e., text vs. voice delivery formats).

In the following, we first introduce related work and propound the hypotheses in Section 2. Then we describe the experimental setup and results of the pilot study in Section 3 and those of the formal study in Section 4. After that, we discuss the findings of both studies and present the design guidelines in Section 5. Finally, we draw conclusions in Section 6.

## 2. Research Background and Our Hypotheses

### 2.1. Feature-Based Explanations

Explanation has long been studied in advice-giving systems (such as decision supports and expert systems; Carenini and Moore, 1998; Chen. and Pu., 2005). In recent years, because of the popularity of recommender systems in various online settings for assisting users' information seeking and decision-making processes, the role of explanation in increasing the system's transparency and user satisfaction has widely been recognized. In an earlier paper about recommendation explanation (Herlocker et al., 2000), the authors compared 21 different interfaces and found that the explanation with grouping of neighbor ratings is the best in terms of convincing users to try the recommended item. However, such collaborative style explanation can cause users to overestimate the item quality, and hence may lead to mistrust or even stop users from using the system (Bilgic and Mooney, 2005).

Therefore, more efforts have been put to leverage the content description of recommended item to provide richer and more informative explanation, so as to allow users to evaluate the recommendation more precisely and comprehensively (Vig et al., 2009; Zhang et al., 2014; Chen et al., 2018, 2019b; Wang et al., 2018a). For this purpose, feature-based explanations have obtained more attention in recent literature (Tintarev and Masthoff, 2015; Chen and Wang, 2017; Kouki et al., 2019; Zhang and Chen, 2020). Specifically, it aims to explain how relevant the currently recommended item is to the user in terms of its features (e.g., “Item X is recommended because it contains feature a, b, …, which are included in items Y, Z, …, that you have already rated”; Symeonidis et al., 2008, p. 1265). Previous studies revealed its advantages against traditional style explanations. For example, Bilgic and Mooney (2005) found that the explanation containing item features helps user estimate the quality of the item more accurately than the collaborative style explanation. Tintarev and Masthoff (2012) showed that users perceive higher satisfaction with the feature-based explanation than the popularity-based explanation (e.g., “This movie is one of the top 100 movies in the Internet Movie Database,” p. 409). Kouki et al. (2019) found that the feature-based explanation is more persuasive than social (e.g., “Your friend Cindy likes …”, p. 380), user-based (e.g., “Customers who bought this item also bought …” Tintarev and Masthoff, 2015, p. 359), and popularity-based explanations.

However, most of existing developments on feature-based explanations have adopted an *ad-hoc* design process. For example, Zhang et al. (2014) designed a simple template such as “You might be interested in [feature], on which this product performs well” (p. 87) to generate the explanation, which was later employed in other works that have focused on algorithmic design (Wang et al., 2018b; Gao et al., 2019; Chen et al., 2020). There is indeed lack of an in-depth empirical investigation on the combined effects of various content design factors of a feature-based explanation on user satisfaction with the recommender system.

To determine key content design factors of a feature-based explanation, we first conducted a literature survey and found that existing explanations mainly vary in terms of the following three aspects (see Table 1): *feature type, contextual relevance*, and *the number of features*. In the following, we introduce each design factor and our corresponding hypothesis.

**Table 1:** Summary of related work on feature-based explanations for recommendations.

**Citation**	**Example of explanation**	**Two-sided**	**Context-relevant**	**No. of features**	**Output modality**	**Product domain**
McCarthy et al. (2005)	*Less memory and lower resolution and cheaper*.	yes	no	3	text	digital products
Symeonidis et al. (2008)	*Article X is recommended because its category is Scientific News and contains the terms global, warming, which are features contained in articles you rated*.	no	no	2+	text	news, movies
Tintarev and Masthoff (2012)	*Although this movie does not belong to any of your preferred genres(s), it belongs to the genre(s): Documentary. This movie stars Ben Kingsley, Ralph Fiennes and Liam Neeson your favorite actor(s)*. Or *This camera cost 679.95 £. This camera is a Nikon. It has an optical zoom of 11.0*x**.	yes	no	2-3	text	movies, cameras
Zhang et al. (2014)	*You might be interested in service, on which this product performs well*.	no	no	1	text	restaurants
Wang et al. (2018b)	*Its decor is neat, good, and nice*.	no	no	1	text	e-commerce, restaurants
Sato et al. (2018)	*Recommend for use “in solitude” for who often visit “noodle.”*	no	yes	0-3	text	restaurants
Chen et al. (2019a)	*They have better values at optical zoom and better opinions at effective pixels, weight, but worse value at price*.	yes	no	4	text	digital product
Kouki et al. (2019)	*U2 is tagged with rock that is in your profile*.	yes	no	1	text	music
Wang et al. (2019)	*Shakespeare in Love is recommended since you have watched Rush Hour acted by the same actor Tom Wilkinson*.	no	no	1	text	movies, music
Li et al. (2020)	*The ramen was delicious*. Or *It is not close to the airport*.	yes	no	1	text	e-commerce, restaurants, hotels
Chen et al. (2020)	*Based on the item you are currently browsing, we recommend you to try this instead because it comes with better battery, screensize, and cord*.	no	no	3	text	e-commerce
Li et al. (2021)	*This product is recommended to you, because its rooftop view is suitable for your current context couples*.	no	yes	1–5	text	restaurants, hotels

#### 2.1.1. Feature Type

The first variation occurs on the type of feature to be explained, for which there are primarily two types: positive (e.g., “delicious food”; Zhang et al., 2014; Pecune et al., 2020) and two-sided (that contains both positive and negative information, e.g., “delicious food, but far distance”; Pu and Chen, 2005; Chen and Pu, 2010; Muhammad et al., 2016). Some studies in recommender systems argued that emphasizing the recommendation's positive characteristic may increase its persuasiveness (Cramer et al., 2008), but some work stated that presenting the two-sided (also called tradeoff-oriented) explanation can help improve users' decision quality (Pu and Chen, 2005, 2007). Therefore, it is still not conclusive how the feature type would actually influence user perception of the explanation's effectiveness, not to mention its interaction effect with other content design factors on users' overall satisfaction with the recommender system.

In our view, if we consider the explanation's main objective is to help users make an informed decision (Schlosser, 2011), its role would be essentially similar to that of a product review. Relevant studies on product reviews demonstrated that presenting a one-sided review can be more useful than a two-sided review (Purnawirawan et al., 2012; Pentina et al., 2018), because during a search process, one-sided argument can help a consumer eliminate or strengthen the position of a product with regards to the alternatives (Chen, 2016). Moreover, evaluative consistency matters when consumers judge whether a review is helpful or not, as inconsistencies between a reviewer's rating and the review content may raise concerns about the reviewer's ability (Schlosser, 2011). As for the recommender system, the inconsistency between its primary function “recommending” and the two-sided message (i.e., including both *pros* and *cons*) may negatively impact users' perceived quality of the recommendation and hence their perceived effectiveness of the associated explanation.

Given this consideration, we hypothesized that the two-sided explanation might have negative influence on users' perceived effectiveness of the explanation (Hypothesis 1).

#### 2.1.2. Contextual Relevance

Contextual relevance refers to whether the explanation indicates the recommendation's relevance to user context such as companion, location, and time, which might be particularly important to the service product because of its contextual sensitivity (Adomavicius and Tuzhilin, 2015; Chen and Chen, 2015; Panniello et al., 2016; Sato et al., 2018; Raza and Ding, 2019). So far there are extensive researches in the area of recommender systems on developing context-aware recommendation algorithms (Adomavicius and Tuzhilin, 2015; Panniello et al., 2016; Raza and Ding, 2019), with the primary focus on using context to increase recommendation accuracy. For instance, it was shown that modeling users' aspect-level contextual preferences as inferred from their reviews can improve the recommendation accuracy (Chen and Chen, 2015). Another research also suggested that incorporating context into the process of generating recommendations can not only increase recommendation accuracy and diversification, but also boost user purchases (Panniello et al., 2016).

However, little attention has been paid to studying the role of highlighting contextual relevance in the feature-based, e.g., “This recommendation's [feature] is suitable for your current [context]” (Sato et al., 2018, p. 659). To the best of our knowledge, only Sato et al. (2018) found that the combination of contextual information with other information (e.g., ratings from similar users, demographics, and item features) in an explanation can improve its persuasiveness and usefulness. Driven by this work, we think that stressing contextual relevance in the feature-based explanation would be likely to make users be more aware of the recommendation's fit with their needs and hence perceive the explanation more effective.

Thus, we hypothesized that providing context-relevant information would be likely to lead people to perceive the feature-based explanation as more effective and hence be more satisfied with the corresponding recommender system (Hypothesis 2).

#### 2.1.3. Number of Features

The number of features in an explanation could also influence users' attitudes toward it. Some related work suggested that the explanation should be concise, focusing only on the most relevant info (Carenini and Moore, 2000), while some stated that the explanation with more features can be more informative and persuasive (Gönül et al., 2006).

According to decision-making and persuasion studies, merely increasing information for a decision can improve the decision maker's confidence (Tversky and Kahneman, 1974), and be perceived more persuasive for enhancing issue-relevant thinking (Petty and Cacioppo, 1984) and serving as a peripheral cue that gives users an impression like “longer messages probably have more or better supporting reasons” (O'Keefe, 2003, p. 252). Several recent studies on online reviews also suggested that longer or more in-depth online reviews can reduce the consumer's uncertainty when evaluating a specific product (Mudambi and Schuff, 2010; Pan and Zhang, 2011).

However, it is not the case that the more features in message, the stronger positive impact it must have on viewers. Explanation with more features may overload the user's limited cognitive resources, so people may tend to skip some information and react faster in response to the long explanation (Fox et al., 2007). Moreover, advertising research also suggested that advertisement with less features can deliver information efficiently, and the relationship between the number of features in advertisement and effectiveness is nonlinear, as when the number of features exceeds a certain value, the positive effects caused by the subsequent content will decrease (Ha and Litman, 1997; Goldstein et al., 2011; Varan et al., 2020).

Based on the previous research, we hypothesized that the explanation containing more features would facilitate users' perceived effectiveness and overall system satisfaction, but there might be diminishing returns (Hypothesis 3).

### 2.2. Output Modality of the Explanation

Note that the above three hypotheses are not independent, but inter-related when all of those three content design factors are considered together. More notably, we think that the output modality of an explanation may interact with those factors to influence user perception. The explanation in the natural language (NL) style is often delivered in two alternative modalities in current systems: *text* and *voice* (for example, text explanations in TripAdvisor and voice explanations in Amazon Alexa). In particular, recent advances in virtual assistants have made the voice output more popular (Kang et al., 2017; Yang et al., 2018; Xiao et al., 2021). The previous persuasion study showed that, compared with texts, the voice messages give people less opportunities to process relevant arguments because exposure to the information is forced rather than self-paced (Petty and Cacioppo, 1986). It was also suggested that written materials could transmit information more efficiently with less distraction, and thus result in greater comprehension and pleasantness compared with audiotaped materials, especially when the materials are complex (Chaiken and Eagly, 1976; Nasco and Bruner, 2008). In the field of human-computer interaction, it was suggested that the speech-based instruction should be short because people find it hard to follow long spoken sentences (Miller, 1956; Sharp et al., 2019).

However, there is no in-depth investigation of how the consuming media would affect user perception of the explanation's effectiveness. In the current research, explanations would become more complex when they include more features. Thus, text explanations would facilitate comprehension as they enable self-paced processing, in which users might be more likely to attend to different information features such as negative features and whether the feature is context-relevant or not. In contrast, voice explanations may hinder the comprehension, as users would be distracted when the explanations contain many features, and be less likely to attend to different information features since the exposure is forced.

Thus, we hypothesized that the effects of the content design factors of a feature-based explanation, i.e., *feature type, contextual relevance*, and *the number of features*, on users' perceived effectiveness and overall system satisfaction would be more pronounced for text explanations, but be less significant for voice explanations (Hypothesis 4).

## 3. Pilot Study

Before exploring how the output modality (i.e., text vs. voice) would interact with those content design factors of a feature-based explanation to affect users' overall satisfaction with a service recommender system, we first conducted a pilot study to investigate how the three factors, i.e., *feature type, contextual relevance*, and *number of features*, when being considered together, would influence users' perceived effectiveness of a feature-based explanation. The pilot study was concretely a 2 (feature type: two-sided vs. pure positive explanations) by 2 (contextual relevance: context-relevant vs. context-irrelevant explanations) by 3 (number of features: 2-feature vs. 3-feature vs. 4-feature explanations) within-subjects design, resulting in totally 12 varied explanation forms presented in the standard plain text format. In particular, in terms of the number of features, most of existing feature-based explanations include one or two features (Zhang and Chen, 2020). As discussed above, we are interested in identifying whether the increased number of features would lead to higher perceived effectiveness, so the number in our study ranges from two (in order to also accommodate feature type such as one positive feature and one negative feature) to four (i.e., price, distance, food quality, and service, as they were found taking major roles in users' decision process when looking for a restaurant; Chen and Chen, 2015). Then, participants read those 12 different explanations about one recommended restaurant and ranked them in terms of their perceived effectiveness. We chose restaurants as the example product domain because they are representative of service recommendations (Zhang et al., 2014; Chen and Chen, 2015; Sato et al., 2018; Wang et al., 2018b; Li et al., 2021).

### 3.1. Participants

We recruited 31 participants (17 females), most of whom were students at the first author's university. Most participants were 20–30 years old (94%), and the others were 30–40 years old (7%). Their education levels included Bachelor (13%), Master (81%), and Doctor (6%). All of them are Chinese. In addition, most participants agreed that they had often looked for recommendation from popular websites/apps when searching for a restaurant (*Mean* = 4.45, *SD* = 0.57, on a 5-point Likert scale: 1 = *strongly disagree*, 5 = *strongly agree*).

### 3.2. Materials and Procedure

All of the participants were presented with the total 12 explanations as described above (see Table 2). Before reading the explanations, participants were asked to vividly imagine the following task vignette^4^ that is typical of a restaurant searching scenario^5^:

Imagining you have a family trip with your parents and your 8-year-old nephew to Florence, Italy. You enjoy the trip and visit some attractions there. After visiting the Piazza della Signoria (the most famous square in the heart of Florence), you are looking for a restaurant to take a family dinner. You prefer to eat some delicious local cuisine such as Italian or Mediterranean food at a mid-range price. Because you walk too much today, you prefer the restaurant within 10 minutes' walking distance.

From now on, the system will recommend to you a restaurant with different explanations. You can also click the restaurant name to view its detailed review information at the TripAdvisor^6^ page.

**Table 2:** (Pilot study) 12 feature-based explanations associated to the same restaurant.

**Two-sided**	**Context-relevant**	**No. of features**	**This restaurant is recommended because**
no	no	2	It has absolutely delicious Italian food within a short walking distance (< 5 min) that you may like.
yes	no	2	It has absolutely delicious Italian food that you may like, but it has relatively high price that you may dislike.
no	no	3	It has absolutely delicious Italian food, friendly service, and within a short walking distance (< 5 min) that you may like.
yes	no	3	It has absolutely delicious Italian food within a short walking distance (< 5 min) that you may like, but it has relatively high price that you may dislike.
no	no	4	It has absolutely delicious Italian food, friendly service, with outdoor seating, and within a short walking distance (< 5 min) that you may like.
yes	no	4	It has absolutely delicious Italian food, friendly service, and within a short walking distance (< 5 min) that you may like, but it has relatively high price that you may dislike.
no	yes	2	It has absolutely delicious Italian food within a short walking distance (< 5 min) that you may like, and the atmosphere is very relaxing for family dinner.
yes	yes	2	It has absolutely delicious Italian food that you may like, and the atmosphere is very relaxing for family dinner. But it has relatively high price that you may dislike.
no	yes	3	It has absolutely delicious Italian food, friendly service, and within a short walking distance (< 5 min) that you may like. And the atmosphere is very relaxing for family dinner.
yes	yes	3	It has absolutely delicious Italian food within a short walking distance (< 5 min) that you may like. And the atmosphere is very relaxing for family dinner. But it has relatively high price that you may dislike.
no	yes	4	It has absolutely delicious Italian food, friendly service, beautiful decoration, and within a short walking distance (< 5 min) that you may like. And the atmosphere is very relaxing for family dinner.
yes	yes	4	It has absolutely delicious Italian food, friendly service, and within a short walking distance (< 5 min) that you may like. But it has relatively high price that you may dislike. In addition, it is good for kids but has no outdoor seating.

*The contextual relevance (e.g., “Its atmosphere is very relaxing for family dinner”) was inferred from customer reviews. The positive/negative sentiments on features were derived from customer ratings (for example, if customers' average rating on “service” is larger than 3 out of 5, it is mapped to “friendly”). The walking distance was estimated via Google map*.

**Figure 1:**
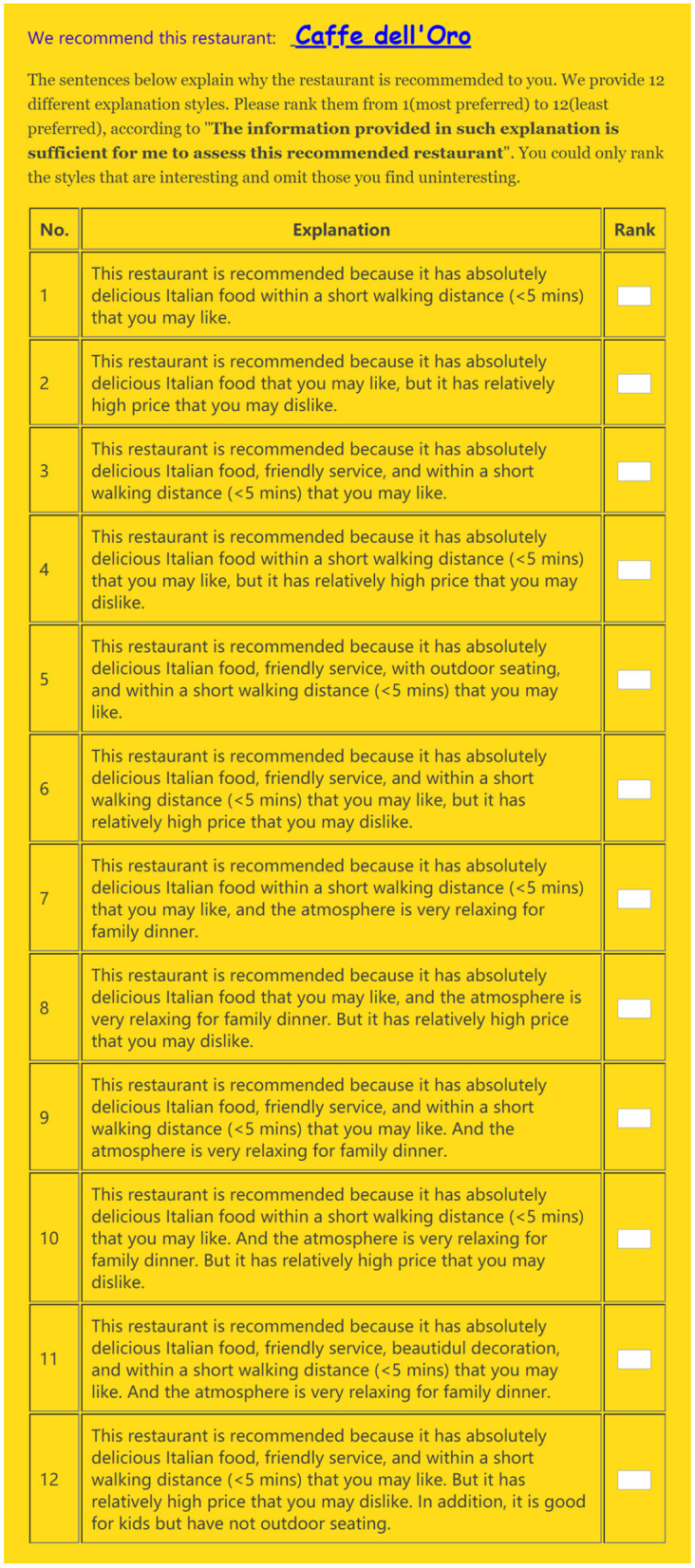
(Pilot study) The display order of all explanations was randomized among participants.

After that, we presented one matching restaurant as retrieved from TripAdvisor, which was accompanied by those 12 different explanations displayed in a random order (see Figure 1). As indicated in the vignette, the recommended restaurant should meet some of the following criteria: family dinner, high food quality, short distance, and middle-ranged price. To manipulate the number of features, we selected two, three, or four features to be shown in the explanation, with three features matching all the requirements for attributes (“high food quality,” “short distance,” and “middle-ranged price”), and four features with “good service” added (though not explicitly mentioned in the vignette). To manipulate the feature type, the pure positive explanation only included the positive feature(s), e.g., “absolutely delicious Italian food,” “friendly service,” and “within a short walking distance (<5 min).” Then, we substituted 1 negative feature for 1 positive feature in the two-sided explanation, e.g., “delicious Italian food, but relatively high price,” given the observation that the occurrence of cons should not dominate that of pros when it is to explain a recommendation (Pu and Chen, 2007). To manipulate the contextual relevance, we included “appropriate for family dinner” in the context-relevant explanation, whereas no such mention in the context-irrelevant explanation. Each explanation was generated via a human-crafted template in order to avoid any mismatches or errors that can be brought by auto language generation algorithms (Costa et al., 2018). The display order of those 12 explanations was randomly decided to eliminate the rank-order effect.

We only showed the restaurant's name at the recommendation page (Figure 1), which was to reduce the confounding effect of other information (such as images, reviews) on users' assessment of the recommendation based on the explanation, but participants were able to click the name to view more details of the restaurant at the original TripAdvisor page.

After reading the 12 explanations, participants were asked to rank them in terms of their **perceived explanation effectiveness**, i.e., “The information provided in this explanation is sufficient for me to assess the recommended item” (1 = *most preferred*, 12 = *least preferred*). We reverse-coded the effectiveness ranking. Higher scores mean higher perceived effectiveness.

### 3.3. Results and Discussion

A three-way repeated measures ANOVA was conducted on users' perceived effectiveness of these 12 feature-based explanations (see Table 3 with descriptive statistics). There was a significant main effect of contextual relevance, *F*_(1, 30)_ = 35.12, *p* < 0.001, $\eta_{p}^{2}$ = 0.54. Participants perceived the context-relevant explanations more effective than the context-irrelevant explanations, suggesting that contextual relevance has positive influence on users' perceived effectiveness of the explanation. In addition, the results showed that there was a significant main effect of the number of features, *F*_(2, 60)_ = 23.14, *p* < 0.001, $\eta_{p}^{2}$ = 0.44. The Bonferroni *post-hoc* test showed that participants perceived the 2-feature explanations less effective than the 3-feature (*M*_*diff*_ = –1.81, *SE* = 0.38, *p* < 0.001) and 4-feature (*M*_*diff*_ = –2.90, *SE* = 0.50, *p* < 0.001) explanations. Furthermore, participants perceived the 3-feature explanations less effective than the 4-feature explanations (*M*_*diff*_ = –1.09, *SE* = 0.40, *p* = 0.034). It reveals that within four features, the perceived effectiveness of explanations increases as more features are provided. Moreover, the main effect of feature type (i.e., two-sided explanation or pure positive) and all the interaction effects among those three content design factors were not significant, *p* > 0.05.

**Table 3:** (Pilot study) Users' perceived explanation effectiveness regarding the three content design factors: feature type, contextual relevance, and the number of features (Mean, and SD in the bracket).

	**Two-sided**			**Pure positive**		
	**2-feature**	**3-feature**	**4-feature**	**2-feature**	**3-feature**	**4-feature**
Context-relevant	5.29	7.87	8.52	6.94	8.29	9.06
	(3.19)	(2.43)	(3.10)	(3.37)	(3.47)	(3.28)
Context-irrelevant	3.58	5.19	6.29	4.23	5.90	7.74
	(3.42)	(3.02)	(3.01)	(3.53)	(3.70)	(3.38)

The results of this pilot study indicate that *contextual relevance* and *the number of features* are two important factors that influence users' perceived effectiveness of feature-based explanations. However, it remains unclear whether these factors may interact with the explanation's output modality (i.e., text vs. voice) to influence user perception, especially their satisfaction with the recommender system. Moreover, multiple explanations for one recommendation may interference each other. Thus, we addressed these limitations in the formal study.

## 4. Formal Study

The formal study extended the pilot study in three ways. *First*, since perceived effectiveness of the explanation can potentially influence user satisfaction with the recommender system (Herlocker et al., 2000; Sinha and Swearingen, 2002; Tintarev and Masthoff, 2012; Zhang and Chen, 2020), we aimed to investigate whether those three content design factors (i.e., feature type, contextual relevance, and the number of features) would further impact the users' overall satisfaction with the recommender system, and whether the effects would be moderated by the output modality of the explanation (i.e., text vs. voice delivery formats). *Second*, in the formal study, we assigned only one explanation to one recommended restaurant to avoid the mutual interference among different explanations. *Third*, we additionally measured the users' behavior of clicking the button “Interested in” or “Not interested in” that is attached to each recommendation, which might further indicate the role of the corresponding explanation in triggering users' interest in the recommendation or not.

### 4.1. Materials and Procedure

The formal study was a 2 (feature type: two-sided vs. pure positive explanations) by 2 (contextual relevance: context-relevant vs. context-irrelevant explanations) by 3 (number of features: 2-feature vs. 3-feature vs. 4-feature explanations) by 2 (output modality: text vs. voice explanations) mixed design. The first three variables are within-subjects factors, and the output modality is a between-subjects factor.

**Figure 2:**
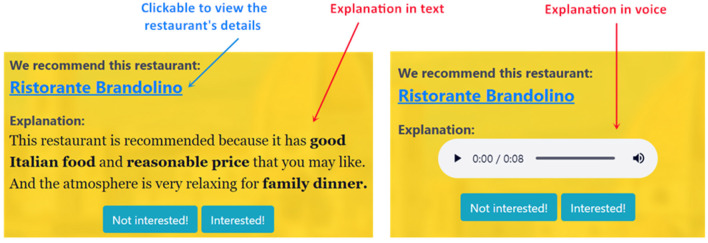
(Formal study) The examples of text explanation (on the left) and voice explanation (on the right).

In either text or voice explanation condition (see Figure 2), participants were first given the task objective below:

Please imagine yourself as the user described in the vignette (similar to that in the pilot study), and then indicate whether the recommended restaurant interests you or not based on its accompanying explanation. The restaurant information used in this experiment was from TripAdvisor.

After that, they were presented with those 12 explanations (see Table 4) delivered either in text or voice (see Figure 2). Being different from the pilot study, the 12 explanations in each condition were randomly associated with 12 different recommended restaurants, respectively, so as to eliminate the potential interference among multiple explanations when users face one recommendation. These restaurants were all from those recommendations by TripAdvisor.

**Table 4:** (Formal study) 12 feature-based explanations respectively associated to 12 different restaurants.

**Two-sided**	**Context-relevant**	**No. of features**	**This restaurant is recommended because**
no	no	2	It has good Italian food and reasonable price that you may like.
yes	no	2	It has absolutely delicious Italian food that you may like, but it has relatively high price that you may dislike.
no	no	3	It has good Mediterranean food, reasonable price, and within a short walking distance (5–10 min) that you may like.
yes	no	3	It has absolutely delicious Italian food within a very short walking distance (< 5 min) that you may like, but it has relatively high price that you may dislike.
no	no	4	It has good Italian food, friendly service, reasonable price, and within a short walking distance (5–10 min) that you may like.
yes	no	4	It has absolutely delicious Mediterranean food, superb service, and reasonable price that you may like, but it is within a relatively far walking distance (15 min) that you may dislike.
no	yes	2	It has good Italian food and reasonable price that you may like. And the atmosphere is very relaxing for family dinner.
yes	yes	2	It has absolutely delicious Italian food that you may like. And the atmosphere is very relaxing for family dinner. But it has relatively high price that you may dislike.
no	yes	3	It has good Mediterranean food, reasonable price, and within a short walking distance (5–10 min) that you may like. And the atmosphere is very relaxing for family dinner.
yes	yes	3	It has absolutely delicious Italian food within a very short walking distance (< 5 min) that you may like. And the atmosphere is very relaxing for family dinner. But it has relatively high price that you may dislike.
no	yes	4	It has good Mediterranean food, friendly service, reasonable price, and within a short walking distance (5–10 min) that you may like. And the atmosphere is very relaxing for family dinner.
yes	yes	4	It has absolutely delicious Italian food, superb service, and reasonable price that you may like, but it is within a relatively far walking distance (15 min) that you may dislike. In addition, it is good for kids but a little noisy.

For each recommendation, only the restaurant's name and its associated explanation were displayed on the interface (see Figure 2). The user could click the name to view the restaurant's details at the original TripAdvisor page. The 12 recommendations were randomly shown one after another.

For participants in the voice condition, they were presented with those 12 explanations in voice (see right of Figure 2). We generated the voice explanation through Amazon Polly's text-to-speech service^7^. This service is based on deep learning technologies to turn text content into voice and to make the speech sound like a human voice. More concretely, we selected *Emma*, a standard British English female voice. In order to put emphasis on the explained feature (e.g., “delicious Italian food”) in the spoken words, we set its volume to “loud' and pitch to “+20%,” and adopted default settings for non-feature words. The voice interface was also plugged with some basic controls such as pause, replay, and drag.

After users evaluated a recommendation based on its explanation, the same question used in the pilot study was asked, i.e., **perceived explanation effectiveness** - “The information provided in the explanation is sufficient for me to assess the recommended item” (Pu et al., 2011). In addition, two more questions were asked because they have popularly been used to assess the impact of explanation on user perception of the recommendation (Pu et al., 2011; Gedikli et al., 2014; Sato et al., 2018; Kouki et al., 2019): **perceived recommendation transparency** - “The explanation helps me understand why the item is recommended” (Pu et al., 2011, p. 161), and **user satisfaction with the recommender system** - “I would enjoy using a recommender system if it presented recommendation in this way” (Kouki et al., 2019, p. 385).

Participants were asked to indicate to what extent they agree with each statement on a 5-point Likert scale (1 = *strongly disagree*, 5 = *strongly agree*). Table 5 shows that the correlations among those three items (i.e., perceived explanation effectiveness, perceived recommendation transparency, and user satisfaction) were all above 0.60 (ranging from moderate to strong strength) at significant level, indicating that if users perceive an explanation effective, it is more likely that they will perceive the corresponding recommendation transparent and be satisfied with the recommender system. The Cronbach's alpha value is 0.84, suggesting a reliable internal consistency (Taber, 2018). Because of the high correlations and reliable internal consistency, we averaged a user's scores on all of the three items to index users' **overall satisfaction** with the recommender system. Higher scores mean higher level of user satisfaction.

**Table 5:** (Formal study) Spearman's correlations among *perceived explanation effectiveness, perceived recommendation transparency*, and *user satisfaction*.

	**Effectiveness**	**Transparency**	**Satisfaction**
Effectiveness (“The information provided in the explanation is sufficient for me to assess the recommended item.”)	1.00		
Transparency (“The explanation helps me understand why the item is recommended.”)	0.68^**^	1.00
Satisfaction (“I would enjoy using a recommender system if it presented recommendation in this way.”)	0.80^**^	0.62^**^	1.00
Cronbach's α = 0.84			

** *Correlation is significant at the 0.01 level (2-tailed)*.

*We used Spearman's correlation because there are univariate and multivariate outliers*.

Beside users' self-reported responses to the above items, we included a measure of users' interest in each recommended restaurant. That is whether they clicked the button “Interested in” (i.e., being interested in the recommended restaurant) or “Not interested in” after they assessed that restaurant's explanation, which may indicate whether the explanation would trigger users' interest in the corresponding recommendation or not. We also included an open-ended questionnaire at the end of this study (i.e., after the user saw all of those 12 recommendations/explanations). Specifically, we asked participants whether they think it is important for them to understand why an item is recommended in such a scenario (i.e., restaurant searching), and if so, what kind of information an explanation should include for assisting their decision making. They were also asked to explicitly specify the number of features they prefer to see in an explanation, as well as their attitudes toward the inclusion of negative feature(s) and contextual information. They were then encouraged to express any other free thoughts and comments. The results are discussed in Section 5.2.

### 4.2. Participants

We recruited 159 participants from Amazon's Mechanical Turk (participation criteria: “Masters,” a qualification granted to workers who have a consistently high performance in the Amazon's Mechanical Turk marketplace; human intelligence approval rate higher than 80%). Each participant was randomly assigned to the text or voice explanation condition. 2$ was paid to the participant if she could complete the whole experiment (mean duration = 16.7 min).

For the results analysis, 38 were excluded because they failed the attention checking questions^8^, took less than 300 s in completing the whole experiment, or gave meaningless answers to the final open-ended questions. Thus, 121 participants remained (56 females), among whom 66 were assigned to the voice explanations and 55 to the text explanations. Most of them were from USA (97) and 20 from India. Their ages span different groups (26 in the range of 20–30, 50 in 30–40, 28 in 40–50, and 17 above 50).

### 4.3. Results and Discussion

#### 4.3.1. Effect on User Satisfaction With the Recommender System

A 2 (feature type: two-sided vs. pure positive explanations) 2 (contextual relevance: context-relevant vs. context-irrelevant explanations) 3 (number of features: 2-feature vs. 3-feature vs. 4-feature explanations) 2 (output modality: text vs. voice explanations) repeated measures ANOVA was conducted on user satisfaction with the recommender system (see Table 6 with descriptive statistics). The results showed that the main effect of contextual relevance was significant, such that participants were more satisfied with the recommender systems providing the context-relevant explanations than those providing the context-irrelevant explanations, *F*_(1, 119)_ = 9.20, *p* = 0.003, $\eta_{p}^{2}$ = 0.07. In addition, the main effect of the number of features was significant, *F*_(1.88, 223.86)_ = 27.80, *p* < 0.001, $\eta_{p}^{2}$ = 0.19. The Bonferroni *post-hoc* test showed that participants were less satisfied with the recommender systems providing 2-feature explanations than those providing 3-feature (*M*_*diff*_ = –0.30, *SE* = 0.05, *p* < 0.001) and 4-feature (*M*_*diff*_ = –0.33, *SE* = 0.05, *p* < 0.001) explanations. However, no significant difference was found between 3-feature and 4-feature explanations (*M*_*diff*_ = –0.04, *SE* = 0.04, *p* = 1.00). The main effects of feature type and output modality were not significant, *p* > 0.05.

**Table 6:** (Formal study) User satisfaction (after aggregation) with the recommender system regarding the explanation's output modality, feature type, contextual relevance, and the number of features (Mean, and SD in the bracket).

		**Two-sided**			**Pure positive**		
		**2-**	**3-**	**4-**	**2-**	**3-**	**4-**
		**feature**	**feature**	**feature**	**feature**	**feature**	**feature**
Voice	Context-relevant	3.87	3.88	4.02	3.99	3.92	4.11
		(0.90)	(0.88)	(0.82)	(0.72)	(0.88)	(0.72)
	Context-irrelevant	4.07	3.95	4.11	4.05	4.00	3.85
		(0.98)	(0.97)	(0.84)	(0.82)	(0.94)	(0.95)
Text	Context-relevant	3.87	4.24	4.24	3.51	4.26	4.27
		(0.85)	(0.58)	(0.52)	(0.93)	(0.57)	(0.62)
	Context-irrelevant	3.47	4.10	3.97	3.17	4.00	4.09
		(0.99)	(0.63)	(0.75)	(0.97)	(0.62)	(0.60)

Further, the results revealed the interaction effect between contextual relevance and output modality, *F*_(1, 119)_ = 16.77, *p* < 0.001, $\eta_{p}^{2}$ = 0.12. In the voice condition, no significant effect was found, *p* > 0.05. In the text condition, participants were more satisfied with the recommender systems providing the context-relevant explanations than those providing the context-irrelevant explanations, *F*_(1, 119)_ = 23.29, *p* < 0.001, $\eta_{p}^{2}$ = 0.16. The results also revealed the interaction effect between the number of features and output modality, *F*_(1.88, 223.86)_ = 30.42, *p* < 0.001, $\eta_{p}^{2}$ = 0.20. In the voice condition, there was no significant effect, *p* > 0.05. In the text condition, users were more satisfied with the recommender systems providing the 3-feature and 4-feature explanations than those providing the 2-feature explanations, *F*_(2, 118)_ = 42.35, *p* < 0.001, $\eta_{p}^{2}$ = 0.42.

Moreover, the results showed the interaction effect between feature type, the number of features, and output modality, *F*_(2, 238)_ = 4.71, *p* = 0.010, $\eta_{p}^{2}$ = 0.04. To investigate the three-way interaction term, we first computed the effect of feature type within each level of combination of the number of features and output modality. In the voice condition, no significant effect was found, *p* > 0.05. In the text condition, participants were more satisfied with the recommender systems providing the two-sided explanations than those providing the pure positive explanations in 2-feature condition, *F*_(1, 119)_ = 11.33, *p* < 0.001, $\eta_{p}^{2}$ = 0.09. But the effects in 3-feature [*F*_(1, 119)_ = 0.14, *p* = 0.71, $\eta_{p}^{2}$ = 0.00] and 4-feature conditions [*F*_(1, 119)_ = 0.66, *p* = 0.42, $\eta_{p}^{2}$ = 0.01] were not significant.

Then we computed the effect of the number of features within each level of combination of feature type and output modality. In the voice condition, there was no significant effect, *p* > 0.05. In the text condition, participants were more satisfied with the recommender systems providing 3-feature and 4-feature explanations than those providing the 2-feature explanations in pure positive condition, *F*_(2, 118)_ = 40.06, *p* < 0.001, $\eta_{p}^{2}$ = 0.40; while participants were more satisfied with the recommender systems providing 3-feature and 4-feature explanations than those providing 2-feature explanations in two-sided condition, *F*_(2, 118)_ = 14.11, *p* < 0.001, $\eta_{p}^{2}$ = 0.19.

**In summary**, this formal study validated that contextual relevance and the number of features influenced user satisfaction with the recommender system. Moreover, we found that the influence was affected by the output modality. In the text condition, participants were more satisfied with the recommender systems providing context-relevant explanations than those providing context-irrelevant explanations, and were more satisfied with the recommender systems providing 3-feature and 4-feature explanations than those providing 2-feature explanations. In addition, participants were more satisfied with the recommender systems providing 3-feature and 4-feature explanations than those providing 2-feature explanations in both pure positive condition and two-sided condition; while participants were more satisfied with the recommender systems providing the two-sided explanations than those providing the pure positive explanations in the 2-feature condition, but not in the 3-feature and 4-feature conditions. In the voice condition, no design factors affected the users' satisfaction with the recommender system.

#### 4.3.2. Effect on User Interest in the Recommendation

To explore the effect of the explanation's design factors on the degree of user interest in the corresponding recommendation (i.e., by clicking the “Interested in” button), we performed the logistics regression for the feature type (0 = pure positive, 1 = two-sided), contextual relevance (0 = context-irrelevant, 1 = context-relevant), number of features [0 = 2-feature, 1(1) = 3-feature, 1(2) = 4-feature], and output modality (0 = text, 1 = voice) on interest (1 = interested in the recommendation, 0 = not interested in the recommendation). The model was significant, χ^2^_(23)_ = 311.71, *p* < 0.001, inferring that this model was better than the null model that did not include the independent variables. Besides, 69% of observations predicted correctly by the model showed an obvious improvement compared to 58% by the null model. Moreover, the model revealed the following significant effects: the effect of feature type, the effect of the number of features, the interaction effect of output modality and feature type, and the interaction effect of output modality and the number of features (see Model 1 in Table 7).

**Table 7:** (Formal study) Significant effects of output modality, feature type, the number of features, and their interaction terms on User Interest in the Recommendation (“Interested in” =1, “Not interested in” = 0) by logistic regression.

**Predictor**	**β**	***SE***	***Wald***	***p***	**Odd ratio**	**95% CI**
**Model 1** (Reference category of No. of feature: 2-feature; Nagelkerke's *R*^2^= 0.26)						
Feature type^a^	–2.82	0.65	18.65	< 0.001	0.06	0.02–0.22
No. of feature			23.64	< 0.001		
No. of feature (3-feature)	1.81	0.47	14.91	< 0.001	6.09	2.44– 15.25
No. of feature (4-feature)	1.96	0.49	16.28	< 0.001	7.11	2.74–18.44
Feature type^a^ × Output modality^b^	2.82	0.74	14.36	< 0.001	16.71	3.90–71.72
No. of feature × Output modality			15.93	< 0.001		
No. of feature (3-feature) × Output modality^b^	–1.68	0.59	8.09	0.004	0.19	0.06–0.59
No. of feature (4-feature) × Output modality^b^	–2.21	0.60	13.53	< 0.001	0.11	0.03–0.36
Intercept	–0.04	0.27	0.02	0.893	0.96	
**Model 2** (Reference category of No. of feature: 4-feature; Nagelkerke's *R*^2^= 0.26)						
Feature type^a^	–2.33	0.49	22.69	< 0.001	0.10	0.04–0.25
No. of feature			23.64	< 0.001		
No. of feature (2-feature)	–1.96	0.49	16.28	< 0.001	0.14	0.05–0.37
Output modality^b^	–1.74	0.47	13.51	< 0.001	0.18	0.07–0.44
Feature type^a^ × Output modality ^b^	2.77	0.61	20.95	< 0.001	16.03	4.89–52.56
No. of feature × Output modality			15.93	< 0.001		
No. of feature (2-feature) × Output modality^b^	2.21	0.60	13.53	< 0.001	9.12	2.81–29.60
Intercept	1.93	0.41	22.65	< 0.001	6.86	

a *Reference category: Pure positive*.

b *Reference category: Text*.

Because the number of features has three levels in our experiment, we not only performed a logistic regression model as shown in the previous paragraph [0 = 2-feature, 1(1) = 3-feature, 1(2) = 4-feature] to identify the difference between 3-feature and 2-feature explanations, and that between 4-feature and 2-feature explanations in the above analyses, but also conducted another logistic regression model [0 = 4-feature, 1(1) = 2-feature, 1(2) = 3-feature] to compare the 3-feature with 4-feature explanations. The second model revealed the same results of Omnibus Test, overall percentage of corrected predictions, and Nagelkerke's R^2 as those of the first model, because the difference between the two models is only the reference group of the number of features. The significant effects found in the second model are: the effect of feature type, the effect of the number of features, the effect of output modality, the interaction effect of output modality and feature type, and the interaction effect of output modality and the number of features (see Model 2 in Table 7).

Since both models indicated the significant interaction effects between output modality and feature type, as well as that between output modality and the number of features, we split the text and voice groups and conducted another two logistic regression models in each group. In the voice condition, the models were not significant, χ^2^_(11)_ = 3.05, *p* = 0.99. It indicated that feature type, contextual relevance, and the number of features of the explanation had no effect on user interest in the recommendation in the voice condition. In the text condition, the models were significant, χ^2^_(11)_ = 295.10, *p* < 0.001 (see Table 8). Besides, the models correctly predicted 78% of observations, indicating a marked improvement than 52% by the null models.

**Table 8:** (Formal study) Significant effects of feature type and the number of features on User Interest in the Recommendation (“Interested in” =1, “Not interested in” = 0) in the Text condition by logistic regression.

**Predictor**	**β**	***SE***	***Wald***	***p***	**Odd ratio**	**95% CI**
**Model 3** (Reference category of No. of feature: 2-feature; Nagelkerke's *R*^2^= 0.48)						
Feature type^a^	–2.82	0.65	18.65	< 0.001	0.06	0.02–0.22
No. of feature			23.64	< 0.001		
No. of feature (3-feature)	1.81	0.47	14.91	< 0.001	6.09	2.44– 15.25
No. of feature (4-feature)	1.96	0.49	16.28	< 0.001	7.11	2.74-18.44
Intercept	–0.04	0.27	0.02	0.893	0.96	
**Model 4** (Reference category of No. of feature: 4-feature; Nagelkerke's *R*^2^= 0.48)						
Feature type^a^	–2.33	0.49	22.69	< 0.001	0.10	0.04–0.25
No. of feature			23.64	< 0.001		
No. of feature (2-feature)	–1.96	0.49	16.28	< 0.001	0.14	0.05–0.37
Intercept	1.93	0.41	22.65	< 0.001	6.86	

a *Reference category: Pure positive*.

Specifically, for the text condition with the 2-feature explanation as the reference group (Model 3 in Table 8), the effect of feature type was significant. Participants who read the pure positive explanations were more interested in the corresponding recommended item than participants who read the two-sided explanations. In addition, the effect of the number of features was significant. In more detail, participants who read the 3-feature explanations or the 4-feature explanations were more interested in the corresponding recommendation than those reading the 2-feature explanations. Furthermore, the main effect of contextual relevance and all the interaction effects were not significant in the text condition when the 2-feature explanation is set as the reference group, *p* > 0.05.

For the text condition with the 4-feature explanation as the reference group (Model 4 in Table 8), the effect of feature type was also significant. It showed that participants who read the pure positive explanations were still more interested in the corresponding recommendation than participants who read the two-sided explanations. In addition, the effect of the number of features was also significant. However, the effect of 3-feature explanations using 4-feature explanations as the reference group was not significant, β = –0.16, *SE* = 0.56, *Wald* = 0.08, *p* = 0.78. It indicates that there was no significant difference between 3-feature and 4-feature explanations as for their effects on user interest in the recommended item. Moreover, the main effect of contextual relevance and all the interaction effects were not significant in the text condition when the 4-feature explanation is set as the reference group, *p* > 0.05.

**In summary**, these results suggest that the explanation's output modality influenced the effects of its feature type and number of features on user interest in the recommendation. In the text condition, pure positive explanations were more effective in triggering user interest in the recommended item than two-sided explanations. Moreover, 3-feature and 4-feature explanations were more effective than 2-feature explanations in this regard. In the voice condition, however, no design factors of the explanation influenced user interest in the recommended item.

## 5. Discussion

### 5.1. Major Findings

Recommender systems have increasingly become an indispensable part for people to make efficient and informed decisions in the era of Internet, in particular in recent advanced virtual assistants based on natural language delivery. Providing explanations to enhance the effectiveness and transparency of recommendations in such application environments is of great importance to potentially increase users' satisfaction with the system. Recently, feature-based explanations have been regarded more useful than other types of explanation, because they provide richer, multifaceted information of the recommended item to facilitate users' decision making (Bilgic and Mooney, 2005; Tintarev and Masthoff, 2012; Chen and Wang, 2017; Chen et al., 2019a; Zhang and Chen, 2020). However, though various forms of feature-based explanations have appeared in existing literature, little work has been done to empirically investigate the combined effects of different content design factors on user satisfaction with the recommender system.

In this research, we have not only examined the effects of three major design factors, i.e., *feature type, contextual relevance*, and *the number of features*, but also evaluated how they interacted with the explanation's output modality (text vs. voice) to influence users' satisfaction with a restaurant recommender system.

Specifically, we conducted two studies. The pilot study was performed in a controlled lab setting, by which we mainly investigated the impacts of feature type, contextual relevance, and the number of features on users' perceived effectiveness of the feature-based explanation. In the formal study, we measured more user perceptions (including perceived recommendation transparency and user satisfaction with the system). Moreover, we tested whether the design factors would influence users' interest in the explained recommendation, as well as how the effects, if any, would be moderated by the explanation's output modality (i.e., text vs. voice delivery formats).

The inter-correlations among the three perception measures (i.e., perceived explanation effectiveness, perceived recommendation transparency, and user satisfaction) indicate that they are strongly correlated with each other, suggesting that if a user perceives an explanation effective, it is more likely that she will perceive the corresponding recommendation transparent and be satisfied with the system that presents recommendation in that way. Because of the high correlations, we averaged a user's scores on all the three items to index the user's overall satisfaction with the recommender system.

There are two major findings from the pilot and formal studies: 1). Of the three design factors, *contextual relevance* and *the number of features* influenced users' perceived effectiveness of feature-based explanations in the plain text output and their overall satisfaction with the recommender system (**Hypotheses 2 and 3 were supported**). Specifically, context-relevant explanations were perceived more effective than context-irrelevant explanations, and participants were more satisfied with the recommender systems providing context-relevant explanations than those providing context-irrelevant explanations. Explanations containing three or four features were perceived more effective than explanations containing two features, and participants were more satisfied with the recommender systems providing 3-feature and 4-feature explanations than those providing 2-feature explanations. 2). Feature type and output modality did not influence users' perceived effectiveness of feature-based explanations and their satisfaction with the recommender system, but it is interesting to see that *output modality* moderated the effects of contextual relevance and the number of features on user satisfaction (**Hypothesis 1 was not supported, and Hypothesis 4 was partially supported**). Specifically, for text explanations (i.e., the explanation delivered in the plain text), participants were more satisfied with the recommender systems providing context-relevant explanations than those providing context-irrelevant explanations, and more satisfied with the recommender systems providing 3-feature and 4-feature explanations than those providing 2-feature explanations. However, no design factors were found to affect user satisfaction with voice explanations (i.e., the explanation delivered in the voice format).

### 5.2. Design Implications

Four design implications are derived from the experimental findings. *First*, in the field of developing context-aware recommendation algorithms, researchers' primary concern is to use context to increase recommendation accuracy (e.g., Adomavicius and Tuzhilin, 2015), with little attention to incorporate context into the explanation (Sato et al., 2018; Li et al., 2021). Extending previous literature, our research demonstrated how context-relevant explanations impact users' perceived effectiveness and overall satisfaction with a service recommender system. In particular, people are more satisfied with recommender systems providing context-relevant explanations than those providing context-irrelevant explanations. However, we did not find the main effect of contextual relevance on users' interest in the recommended item, which implies that, though it may help increase the explanation's effectiveness from users' perspective, it may not necessarily trigger users' interest in the recommendation itself. In the final open-ended questionnaire, some participants mentioned the role of context-relevant explanations in helping them decide whether the recommendation is a right choice or not, e.g., “To decide which is best suitable for me at the current situation.” (P_13) “It is important for me to understand why items are recommended in this scenario, because I need to know lots of different factors to make my final decision.” (P_79) “I enjoyed the family aspect, but not necessary for my decision unless I have kids” (P_117).

**Design implication 1:** Explaining the relevance of a service recommendation's features to user context can be helpful for increasing the explanation's effectiveness and user satisfaction with the recommender.

*Second*, previous literature contradicts on how length impacts effectiveness of the feature-based explanation. In current research, we investigated this issue by in-depth comparing the effects of explanations containing two, three, and four features respectively. We consistently found that 2-feature text explanations were less effective than text explanations containing three or four features. Participants were more satisfied with the recommender systems providing 3-feature and 4-feature text explanations than those providing 2-feature text explanations. No conclusions could be drawn to the difference between 3-feature and 4-feature text explanations. It thus suggests that in terms of increasing explanation effectiveness and user satisfaction, text explanations containing three or four features can perform better than those with two features. However, our findings did not reject the information overload assumption (Fox et al., 2007). It still remains unclear whether users' perceived explanation effectiveness and overall satisfaction with the recommender system would increase when the maximal number of contained features is beyond four.

In addition, in the final questionnaire, participants expressed different opinions on the number of features. Several participants preferred to see more details, e.g., “I need a lot of details. Like exactly how far away and how much price range.” (P_47) “The length of the review is not a negative. I would rather have too much information rather than too little, especially if I'm the one who is looking for many particular things.” (P_66) “Some of the explanations were very perfunctory (very short) they did not have a lot of detail and it was difficult to make a decision.” (P_95) “I really enjoyed the longer descriptions that gave me more information, even if it wasn't what I was looking for” (P_32) “the more information provided, the better” (P_89). Few participants considered that long explanations might cause information overload, e.g., “There is a thing as too much information, but I don't think any of these recommendations hit that line.” (P_82) “Taking into account every type of situation is helpful, but can get lengthy” (P_86).

Therefore, we think future research should be done to ideally determine the turning point via more empirical studies. Moreover, in both studies participants were instructed to find a restaurant for a family dinner, providing delicious local cuisine, at a mid-range price, and within 10 min' walking distance. These requirements may influence how the number of features impacted participants' perceived effectiveness of the explanation as well as their overall satisfaction, since 3-feature or 4-feature explanations might intuitively perform better in meeting these requirements than 2-feature explanations. However, we did not find significant difference between 3-feature and 4-feature explanations, which might infer that the impact of the number of features on explanation effectiveness and user satisfaction did not covary with the task requirement. Future research should investigate how the number of features impacts explanation effectiveness by eliminating the interference of task requirement.

**Design implication 2:** Users generally prefer to see three or four features in the explanation, but including more features might cause information overload.

*Third*, most of current feature-based explanations focus on pure positive explanations. Although some work proposed the so-called two-sided (or tradeoff-oriented) explanations containing both positive and negative features (Pu and Chen, 2007; Chen and Pu, 2010), little research has empirically investigated how the feature type (e.g., two-sided vs. pure positive) impacts effectiveness of the feature-based explanation. In current research, we fill this gap by finding no significant effect of feature type on the explanation's effectiveness, except that in the formal study, participants showed significantly lower interest in the recommended item when they read the two-sided text explanations than the pure positive text explanations. Previous marketing research suggested that people prefer messages containing both positive and negative information (e.g., Kamins et al., 1989), because people may think that the two-sided messages are honest. Previous research in recommender systems also showed that providing the two-sided explanation (containing both pros and cons) can help induce users' trust in the system (Pu and Chen, 2005, 2007). Muhammad et al. (2016) showed that the explanation containing both pros and cons features can help improve the explanation's overall clarity and helpfulness. However, as our results show, it does not mean that users will be more interested in the recommendation with such explanation.

Nevertheless, we found that participants were more satisfied with the recommender systems providing the two-sided explanations than those providing the pure positive explanations in the 2-feature condition, but not in the 3-feature and 4-feature conditions. In the final questionnaire, participants expressed different opinions. Some would like to see the two-sided explanations, e.g., “I want to see 1 positive review and 1 negative review on each explanation page, too.” (P_48) “I like that it doesn't just show exact matches. If a restaurant meets 4/5 of my criteria, I might pay a little more or walk a little further to get there. I would need to be able to mark some criteria non-negotiable though” (P_120). These comments imply that the two-sided explanation might be useful for users to make a more informed, trade-off decision, especially when there are only two features involved. However, some of other participants said that they would like to know what features led the recommendation to satisfy their requirements, so they actually did not expect to see negative information in the explanation, e.g., “If even one need is not met then my family will not want to go there. I need to know just what factors led to the recommendation to be sure all requirements were met” (P_60). Future research should further explore this issue, for example, to study the moderating effect of users' personal characteristics, e.g., decision styles (Henderson and Nutt, 1980), on their preference for the two-sided explanation, and to take into account the recommender system's objective as well, e.g., to persuade users to accept the recommended item (Kouki et al., 2019) or to increase their trust (Pu and Chen, 2005, 2007).

**Design implication 3:** Including negative feature(s) in the explanation (i.e., two-sided with both *pros* and *cons*) does not help to increase its effectiveness, but it may support users to make tradeoff if only two features are involved.

*Fourth*, contrary to previous suggestion that voice messages limit information processing and comprehension (Petty and Cacioppo, 1984), we found no significant difference between text and voice explanations. It might be because our explanations were not complex. Preece et al. (2015) suggested that speech-based instruction should be short because people find it hard to follow long spoken sentences. Similarly, Chaiken and Eagly (1976) suggested that the negative effect of voice on information processing is more pronounced when the materials become complicated. Moreover, people's motivation might also explain the non-significant findings. People turn to recommender systems to help them choose an item that best meets their requirements, which might not require deep-processing. Consistent with this suggestion, we found that the main effects of contextual relevance and the number of features on users' perceived explanation effectiveness and overall satisfaction with the recommender system were only found in text explanations but not in voice explanations. What factors may affect users' perceived effectiveness of voice explanations may need further investigation. The concerns as expressed by some participants about voice explanations in the final open-ended questionnaire also support us to further explore, e.g., “The voice was a little hard to listen to.” (P_23) “It was difficult to find what I was looking for it on a vocal basis” (P_39) “I think the voice is too slow. I think quicker speech would be easier to trust and listen to.” (P_47) “I thought the voice sounded a bit strange and alien like.” (P_49) In the future, it is important to investigate how the changes on voice variables (e.g., rate, gender, volume, and pitch) would influence user perception.

**Design implication 4:** The effects of content design factors of a feature-based explanation on user satisfaction are found more pronounced for text explanations, but less significant for voice explanations. For the latter, as lengthy and complex information may hinder users' comprehension of the spoken message, short and simple explanation would be preferred.

## 6. Conclusion

To the best of our knowledge, this is the first work that empirically evaluated the combined effects of various content design factors about feature-based explanations, as well as their interaction effects with the explanation's output modality (text vs. voice) on users' satisfaction with a service recommender system. In particular, we found that two design factors, i.e., *contextual relevance* and *the number of features*, interact with *output modality* to influence user satisfaction with the system. Specifically, for text explanations, context-relevant ones behave more effectively than context-irrelevant explanations, and explanations containing three or four features are more effective than explanations containing two features. In consequence, users are more satisfied with the recommender systems providing context-relevant explanations than those providing context-irrelevant explanations, and more satisfied with the recommender systems providing three or four features than those providing two features. However, these significant results are not valid for voice explanations, which might be because, when the explanation becomes complex (e.g., containing more features or context-relevant information), the voice output may hinder users' comprehension and hence be not easy for them to attend to different information features. Near the end, we derived four design implications based on these experimental findings and users' free comments, which in our view could be constructive for designing more effective feature-based explanations for service recommender systems.

There are two major limitations of this work. *Firstly*, the studies were limited to restaurants, so the generalization to other service products (e.g., hotels) and even non-service domains (e.g., music, movies, and e-commerce) might need to be further validated. *Secondly*, users were asked to interact with pre-prepared explanations for an imagined task scenario, which might not fully reflect their reactions toward a real recommender system. In the future, we will be engaged in addressing these limitations through more studies. Moreover, we will be interested in conducting in-depth investigation on voice explanations by considering some speech-specific variables (e.g., rate, gender, volume, and pitch).

## Data Availability Statement

The raw data supporting the conclusions of this article will be made available by the authors, without undue reservation.

## Ethics Statement

The studies involving human participants were reviewed and approved by Research Ethics Committee, Hong Kong Baptist University. The patients/participants provided their written informed consent to participate in this study.

## Author Contributions

ZZ and LC designed the two studies. LC, TJ, and YL contributed to the hypotheses. ZZ performed the pilot study. LL performed the formal study. ZZ, LC, and TJ contributed to the data analyses and wrote the article with inputs from other authors. All authors contributed to the article and approved the submitted version.

## Funding

This work was supported by two research grants: Hong Kong Baptist University IRCMS Project (IRCMS/19-20/D05) and Hong Kong Research Grants Council (RGC/HKBU12201620).

## Conflict of Interest

The authors declare that the research was conducted in the absence of any commercial or financial relationships that could be construed as a potential conflict of interest.

## Publisher's Note

All claims expressed in this article are solely those of the authors and do not necessarily represent those of their affiliated organizations, or those of the publisher, the editors and the reviewers. Any product that may be evaluated in this article, or claim that may be made by its manufacturer, is not guaranteed or endorsed by the publisher.

## Footnotes

^1^ https://www.amazon.com/b?node=19182213011

^2^ https://support.apple.com/en-hk/guide/iphone/iph6f94af287/ios

^3^The experimental procedure of both studies was reviewed and approved by the University Research Ethics Committee.

^4^The reason we asked all participants to imagine the same task scenario was to avoid noises arising from natural variances associated with individual differences. This approach has been commonly adopted to evaluate information display in the area of human-computer interaction (Herlocker et al., 2000; Kitchener and Jorm, 2002; Chen and Pu, 2010; Jin et al., 2019; Ngo et al., 2020).

^5^The vignette was created based on our reading of over 100 restaurant reviews at TripAdvisor, so as to cover some representative criteria users have when they ask for restaurant recommendations.

^6^https://www.tripadvisor.com/Restaurants, which is a popular traveling website that provides recommendations (e.g., about restaurants, hotels, and travel experiences) based on user-generated content.

^7^https://aws.amazon.com/polly/.

^8^We repeated two attention check questions for users to answer each twice. Participants who answered inconsistently were considered as failing attention check.
